# Application of Post-Industrial Leather Waste for the Development of Sustainable Rubber Composites

**DOI:** 10.3390/polym17020190

**Published:** 2025-01-14

**Authors:** G. Barrera Torres, Carlos M. Gutierrez Aguilar, Elizabeth R. Lozada, Manuel J. Tabares Montoya, Beatriz E. Ángel Álvarez, Juan C. Sánchez, Jaime A. Jaramillo Carvalho, Renivaldo J. Santos

**Affiliations:** 1 Faculty of Arts and Humanities, Instituto Tecnológico Metropolitano (ITM), Medellín 050036, Colombia; eliza.rico.lozada@gmail.com; 2 School of Engineering, Universidad Pontificia Bolivariana (UPB), Medellín 050031, Colombia; manuel.tabares@upb.edu.co (M.J.T.M.); beatriz.angel@upb.edu.co (B.E.Á.Á.); 3 Advanced Manufacturing Technology Center, Servicio Nacional de Aprendizaje, Medellín 050036, Colombia or juan.sanchezg@pascualbravo.edu.co (J.C.S.); jaime.jaramilloc@pascualbravo.edu.co (J.A.J.C.); 4 Mechanical Department, Institución Universitaria Pascual Bravo (IUPB), Medellín 050036, Colombia; 5 Faculty of Engineering and Science, Sao Paulo State University (UNESP), Rosana 19274-000, SP, Brazil; renivaldo.santos@unesp.br

**Keywords:** leather waste, natural rubber, rubber composites

## Abstract

The substantial waste generated during the processing of hides and skins as well as at other stages of manufacturing is a recurring issue in the leather industry that this article attempts to address. To investigate the mechanical and thermal characteristics of the resultant composites, this study suggests using leather waste from the bovine leather industry, analyzes the tanning process, and assesses the viability of mixing this waste with natural rubber (TSR-20). Without the inclusion of leather waste, the resulting composites had exceptional tensile strength, surpassing 100% of rubber’s strength. The effective interaction of the recycled leather particles with the natural rubber matrix was evidenced using the Lorentz–Park equation. This better performance points to a competitive relationship between rubber and leather waste. The samples’ density was 10% greater than that of polybutadiene elastomers and 10% greater than that of natural leather, while the hardness was comparable to that of PVC, which is frequently utilized in the design of general-purpose soles. This suggests that waste from the leather industry can be efficiently utilized in sustainable applications, particularly in the production of leather goods and shoes, helping to valorize waste that is typically discarded. Furthermore, by encouraging the use of recycled resources in the creation of new compounds, this plan provides the rubber sector with a sustainable option. To optimize this proposal, perhaps will be necessary to identify different vulcanization systems to improve the physical mechanical properties and other uses derived from the optimizations realized. This composite can be applied in the fashion industry in order to develop new trends around the application of waste and residues for a natural design line. Through the research process, it was possible to integrate the residues into the natural rubber matrix, as evidenced in the characterization process.

## 1. Introduction

Industrial processes aim to obtain products by transforming raw materials, which often requires the use of natural resources. In recent decades, growing concerns about pollution and climate change have drawn attention to the way these resources are used [1]. The industrial sector, one of the major contributors to these problems, is becoming increasingly focused on reducing resource consumption and pollutant emissions. An example is the bovine leather manufacturing and processing sector, usually referred to as tanning. Several studies [2,3,4,5] have proposed alternatives to mitigate environmental impact in this industry; however, they remain insufficient. This problem is also observed in the Colombian tanning industry, which lacks programs such as those aligned with the 2030 Sustainable Development Agenda, responsible consumption, and climate action [6,7,8]. To promote sustainable production and manufacturing and the consideration of global factors in the process, the manufacturing industry could support the fulfillment of sustainable development goals by seeking to reduce the emission of waste by incorporating it into the production cycle rather than dumping it in landfills.

In the global context, the United Nations Environment Program (UNEP) introduced in the 1980s the concept of Cleaner Production as a strategy to reduce the excessive consumption of natural resources and make production processes more efficient [9]. The main objective of such initiatives was to reorient industrial processes, products, and services towards preventing environmental impacts. Examples include the treatment of contaminated water [10,11,12]. Furthermore, global leather supply chains constitute a socio-technical system, consisting of stakeholders, such as consumers, civil society, and scientific communities; contextual factors, such as demand, product requirements, production, transportation, and communication; and social mechanisms, such as collaboration and working conditions [13].

Around the world, various industries are implementing regulations to support sustainability, and some businesses are adopting more conscientious production methods. However, some sectors continue to rely on outdated and environmentally harmful technologies instead of taking preventative measures [14,15]. The tanning industry has faced challenges due to its reliance on traditional processes for treating animal skins. Consequently, research is exploring alternatives to the conventional chrome tanning process. Although the leather tanning industry has seen minimal changes in its core production processes, increasing demand has driven efforts to automate certain stages and incorporate new technologies to improve production efficiency [16,17].

The leather industry generates solid waste categorized into non-tanned and tanned waste. Non-tanned waste includes by-products from fleshing, cutting, trimming, and sanding operations. After tanning, semi-finished and finished leather shavings and sawdust are also generated. Leather waste from the tanning process takes over 100 years to biodegrade, making it one of the least valued and most problematic wastes in the industry. Moreover, out of 1000 kg of raw hide, nearly 850 kg becomes solid waste in tanneries, while only 150 kg is transformed into leather. In other words, although part of this material is used in the manufacturing of leather goods, most ends up in public landfills [18].

A global study revealed that China leads in leather production, followed by Italy, Vietnam, and Germany. India’s share is 2.7%, worth USD 1.4 billion. The largest importers are the USA, Germany, and EU countries. Despite its economic significance, leather production negatively impacts the environment [19]. The study also highlights the presence of tanneries in 11 departments in Colombia, and the country’s leather industry is considered a key production sector in the National Development Plan, with trade surpluses recorded since 2003.

This industry is particularly important in the footwear and leather goods sectors [20]. In the case of Colombia, the footwear sector experienced a 13.3% increase in exports between 2017 and 2018, with a 4.73% rise in real annual spending on footwear purchases by November 2018. This underscores the importance of adopting alternative waste management strategies in footwear production [21]. Efforts have been made to repurpose leather waste as a reinforcement phase in the production of rubber-based materials. For that reason, this study focuses on combining leather residue (LR) with natural rubber (NR), considering its excellent physical, mechanical, and chemical properties, which offer an environmentally friendly material. As a polymer, natural rubber allows for the incorporation of particles and fibers, enhancing its properties for new industrial applications [22,23,24,25].

Furthermore, environmentally friendly composites—also known as green composites, ecological composites, or biocomposites—show physical, mechanical, thermal, and chemical characteristics comparable to traditional materials but with the added benefit of environmental sustainability [26,27]. Natural rubber, as a sustainable material, is increasingly being used in new developments. In fact, over the past six years, the global production of polymers from sustainable natural sources has increased by more than 400% [28].

Given their favorable technical qualities, affordability, and environmental attributes, rubber composites reinforced with plant and animal fibers are being used in numerous industries [29]. Moreover, the mechanical properties of reinforced natural rubber composites, such as strength and modulus, elongation at break, creep resistance, increased toughness, and shear and tear strengths, are markedly superior to those of particulate-reinforced rubber [30].

As a result, a variety of products have been developed in Colombia using these composites, including conveyor belts and footwear [31]. This has been achieved through a strategic alliance with the Colombian National Training Service (abbreviated SENA in Spanish), which provides the necessary infrastructure to formulate novel proposals on the use of leather residues in the rubber industry and introduce them to the productive sector. Consequently, this study aims to evaluate the technical feasibility of producing blends of natural rubber and leather residues for use in footwear products.

The methodologies consulted for this project include steps for ensuring efficient and sustainable production in the leather tanning industry (see Figure 2). A comparison was made between the technical information found and the documentation applicable to the Colombian industry, aiming to identify the benefits of implementing reuse alternatives for leather waste. These efforts contribute to mitigating the environmental damage caused by improper disposal of leather waste while proposing viable solutions for the development of new products within the leather industry [32,33,34,35].

**Figure 1:**
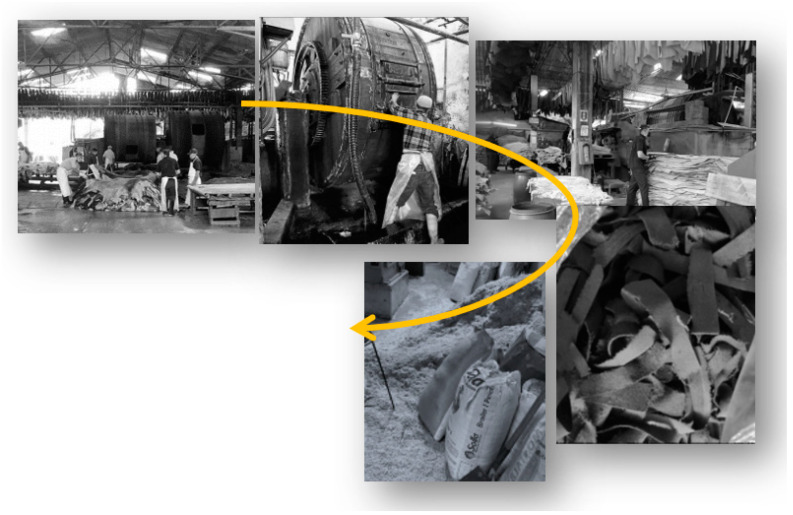
From left to right: reception of skins, tanning drum, drying, residues. Source: Authors’ own work.

The production model around waste use focuses on reducing the environmental impact of production activities through comprehensive preventive measures. It emphasizes resource reduction by adopting advanced process technologies and equipment, thereby achieving sustainable development [36]. Figure 1 illustrates each stage of the tanning process, which proceeds in the following order: pre-treatment (receiving, pre-fleshing, liming, fleshing, and splitting), tanning (dewatering, trimming, dyeing, and drying), and post-treatment (conditioning, finishing, packaging, and shipping).

One of the least utilized residues throughout the production process is post-industrial leather, generated after the cutting of pieces. This residue is typically discarded in municipal dumps without any probability of use, which is why it was selected as the focus of this study.

**Figure 2:**
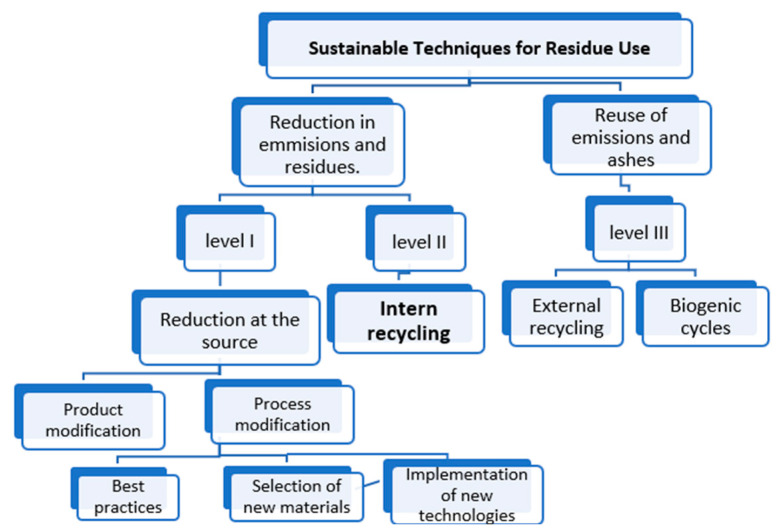
Typical sustainable production process [37].

The study identified several processes in which this waste could be reprocessed. Figure 2 illustrates some potential alternatives, where the decision was made to reduce post-industrial leather waste by adopting the Level II strategy—internal recycling of waste. This strategy involves reusing residues by mixing them with a binder, in this case, natural rubber. The implementation of this approach holds great promise, especially for small entrepreneurs, as the potential for designing new products with this composite material is highly feasible and within their technological reach.

Figure 2 presents three levels of sustainable production methods, which serve as mitigation alternatives to reduce the environmental impact of production processes [38,39]. These levels represent strategies for optimizing factory operations. Level I involves actions at the product or process level aimed at reducing or eliminating waste generation. This includes improvements in the production process, the substitution of raw materials with less polluting or more reusable alternatives that generate fewer by-products, or the adoption of more efficient technology. Level II focuses on internal recycling actions, where by-products and waste are reintegrated into the production process to maximize resource use. Finally, Level III encompasses recycling actions that are external to the company; i.e., waste generated within the company is repurposed as raw material for other industries. This study explored the Level II alternative, as previous research has demonstrated the potential of developing synthetic leather by combining it with natural rubber to create a useful composite for the production sector [40].

The methodology for rubber conformation used in developing the mixtures follows the process described in [21,29,41,42]. First, the mixtures of leather residues and natural rubber were analyzed and characterized. This process began by identifying the residues generated during production, followed by an analysis of their functionality when mixed with a binder such as natural rubber, aiming to project potential industrial applications. To prepare NR mixtures with leather waste, vulcanization activators were added to a roller mixer, and proportional calculations were made per hundred parts of rubber (phr). The mixture was processed at room temperature for 6 min to achieve the plasticization of the elastomer. The leather residue was then added, and the mixing process continued until the material was completely dispersed.

## 2. Materials and Methods

**Figure 3:**
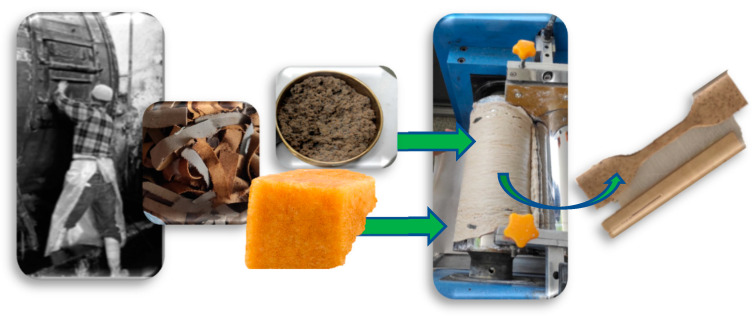
From left to right: skins on tanning drum, milled residues, mixing process residues with natural rubber in an open roller mixer, and samples obtained for the characterization process. Source: Authors’ own work.

The leather residues were initially shredded using an industrial shredder and left to dry at 80 °C for 24 h. The dried material was then sieved using a Ro-Tap sieve shaker W.S. Tyler®—ro-tap Shaker, passing through a 30-mesh sieve with an aperture size of ≤600 μm, after which the sieved material was reserved for further use. Composites of natural rubber and finished leather particles were prepared with varying concentrations of leather particles at 10%, 20%, 30%, 40%, 50%, and 60% phr; the process is shown in Figure 3. Once the leather fibers were mixed and evenly dispersed in the natural rubber, vulcanization activators were introduced. The composite was then mixed in a two-roll rubber mill at 60 °C for 20 min, with a friction ratio of 1.0:1.25. The leather fiber ratios were selected based on previous studies involving wet blue leather residues.

The activated mixtures were stored at room temperature for 24 h to allow for sufficient reaction time and to eliminate residual gasses, according to ASTM 3182 [43]. The formulations used in this work are shown in Table 1. Initially, the natural rubber was chewed for 10 min, so that a band would form around the roller and there would be a momentary loss of the elastic characteristic through the shearing of the polymer chains, making it plastic with low viscosity, facilitating the incorporation of filler and reagents and also their reaction with the rubber. After being chewed and plasticized, the reagents and filler were inserted until they were fully incorporated. The zinc oxide and stearic acid were incorporated simultaneously, followed by the leather residue until they were completely homogenized.

This facilitated the action of the accelerators and sulfur that would be added after this period. After the accelerators and sulfur were added, the compound was left to rest for another 12 h at room temperature so that the reaction between the zinc stearate and the accelerators could take place. In the next step, the composites were subjected to rheometric testing (ASTM D 5289) [44] to determine the optimum vulcanization time (T90) used in thermo-pressing, maximum torque (MH), and minimum torque (ML). The composites were molded by compression in a hydraulic press at a temperature of 150 °C for times equal to T90. After thermo-pressing, the composites obtained at different phr were subjected to mechanical, thermal, and morphological tests. In this final stage, the physical, mechanical, and thermal properties of the mixtures were evaluated in accordance with the international standard ASTM D 3184-07 [45].

### Rheological Properties

To gain insight into the vulcanization behavior, rheometry experiments were conducted using a rheometer (Team Equipment Ltd., Sao Pablo, Brazil) with an isothermal system set at 150 °C and a disk oscillation of 1°. The curing parameters were determined based on the curves obtained in accordance with ASTM D2084 [46]. Among the rheological properties, viscosity and elasticity are key factors related to structural characteristics such as weight and molecular weight distribution. In the case of blends, molecular weight distribution and phase morphology are particularly important, as the overall process outcome depends on the understanding of these variables. Studying the mixture’s characteristics allows for the observation of polymer conditions, including degradation and crosslink formations, which manifest as a reduction or increase in torque.

The swelling method was employed to ascertain the crosslinking density of the composites. The samples, with a mass of approximately 0.25 ± 0.05 g each, were first weighed and then immersed in toluene for 5 days. After this period, the specimens were removed, surface-dried with absorbent paper, and weighed again. They were then placed in an oven at 60 °C for 24 h and weighed one final time. The crosslinking density was calculated using Equation (1), as suggested in [47,48].

(1) $$\eta =\frac{-(Ln\left(1-V_{b}\right)+V_{b}+X(V_{b}{)}^{2})}{\left(p_{b})(V_{0})(V_{b}^{\frac{1}{3}}-\frac{V_{b}}{2}\right)}$$

Here, *V_b_* is the volume fraction of the polymer in the swollen gel at equilibrium; *X*, the polymer–solvent interaction parameter; *P_b_*, the density of the polymer; and *V*_0_, the molar volume of the solvent. The relative density was determined using a Sartorius BA210® gravimeter, and the densities of the various samples were measured following the specifications outlined in the UNE-ISO 2781:2015 Method A standard [49].

The mechanical properties of the composites were evaluated through tensile and deformation tests, including calculations for Young’s modulus and elongation at break. Measurements were conducted using universal analysis equipment in accordance with ASTM D 412-06a [50]. The tests were performed using a C-type mold, with a displacement speed of 500 mm/min. In addition, abrasion tests were conducted in triplicate using a rubber abrasion tester featuring a revolving drum, in accordance with ASTM D 5963 [51]. The samples were subjected to a friction distance of 40 m, equivalent to 84 drum rotations, under a force of 5 ± 0.2 N (1.125 − 0.02 lbf).

Hardness tests were performed using a Kiltler durometer (Sao Pablo, Brazil) with a Shore A scale, in accordance with ASTM D 2240 [52]. Mass loss measurements were obtained through thermogravimetric analyses, which also provided insights into the thermal stability of the materials—a critical property for this study. Analyses were carried out on a TA Instruments Q500 analyzer, covering a temperature range between 25 °C and 500 °C. It is important to clarify that the thermal degradation behavior of collagen fibers varies depending on the modifications applied to the fibers from different hides, due to their inherent structural complexity [53].

The derivative thermogravimetry (DTG) curve served to determine the temperature at which material loss occurred. Scanning electron microscopy (SEM) was employed to further evaluate the interaction between the constituents, specifically regarding morphology, homogeneity, and dispersion. Moreover, the energy-dispersive X-ray (EDX) technology integrated into the SEM was used to identify regions in the images that could be associated with chemical elements that did not react during the vulcanization process or flaws in the physical dispersion of the mixture components. When the electron fringe is incident on the composite, the outermost electrons are exited, causing changes in energy levels. As these electrons return to their original state, energy is released and captured by the detector. Since each chemical element has a unique energy spectrum, it can be accurately identified. The equipment used for this analysis was the Philips® XL-30 FEG microscope, equipped with energy-dispersive spectroscopy (EDS).

## 3. Results

In the preparation of the natural rubber composites, oleic acid—a long-chain unsaturated fatty acid derived from animal and vegetable fats through saponification and acidification—was added after incorporating the leather fibers during open roller milling [54]. This addition aimed to improve the interaction between the fibers and the natural rubber, thus promoting good dispersion and enhancing the physical and mechanical properties of the composites.

### 3.1. Rheometry and Crosslinking Density of Composites

**Figure 4:**
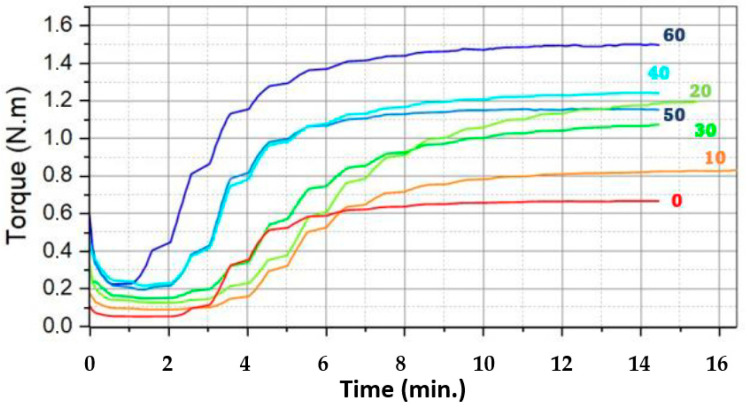
Rheological behavior curves for NR/LR composites.

To gain a more detailed understanding of the variables influencing the characteristics of the composites compared to vulcanized rubber without fillers, the rheometric parameters are presented in Table 2. Notably, the optimum cure time (t90) gradually increases for samples containing 10 to 20 phr of leather residues (see Figure 4). This can be attributed to the proportion of fibers added and the acidic nature of leather, which inhibits the activity of the vulcanization system accelerators. The most salient characteristic is the linear increase in torque, which correlates with the addition of leather fibers to the samples.

The gradual increase in optimum cure time for samples with 10 to 20 phr of leather residues can be attributed to the acidic nature of the leather residues. This acidity inhibits the action of the vulcanization system accelerators, as these interact with the free functional groups of the leather [55,56]. However, this inhibition results in improved physical and mechanical properties of the composite, which is analyzed in subsequent sections.

For samples containing 30 to 60 phr of residue filler, a gradual reduction in cure time was observed, with the 60% leather residue sample showing a shorter cure time compared to pure rubber. These results suggest that the addition of higher filler proportions promotes the vulcanization processes, likely due to the active groups present in the leather, as suggested in [57]. These findings are consistent with those of crosslinking density analyses, where a shorter cure time indicates a faster reaction rate and reduced crosslinking production time [58].

The minimum torque (ML) values were observed during the pre-cure stage, when the accelerators and zinc stearate initiated the reaction process but no crosslinking had yet occurred. ML helps determine the degree of processability, i.e., the viscosity of the unvulcanized composites [59]. In contrast, the maximum shear strength (MH) values are associated with material stiffness. An increase in MH in the rheometer’s oscillating disk may suggest an increase in matrix stiffness, necessitating greater rotational stress.

A gradual increase in minimum and maximum torque values was observed for all samples containing residues. This indicates that the viscosity and stiffness of the composites increase with the incorporation of leather residues, which are inherently stiffer due to their fibrous structure. The increase in MH can be attributed to the reduced macromolecular mobility of the matrix resulting from the incorporation of leather fibers [60]. The slight reduction in maximum torque for the composites with 30 and 50 phr can be attributed to the limited amount of chemicals available for vulcanization, as reported in [56]. The maximum torque values were considered together with the results of the hardness analyses, which will be discussed below. The obtained values are of great importance because they orientate the possibilities of saving in the cost of equipment in vulcanizing; considering some similar investigations, where the T90 value was obtained after 16 min of processing with NBR rubber [61], in this research, the T90 value was favorable as it was less than 12 min.

### 3.2. Determining Crosslinking Density

**Figure 5:**
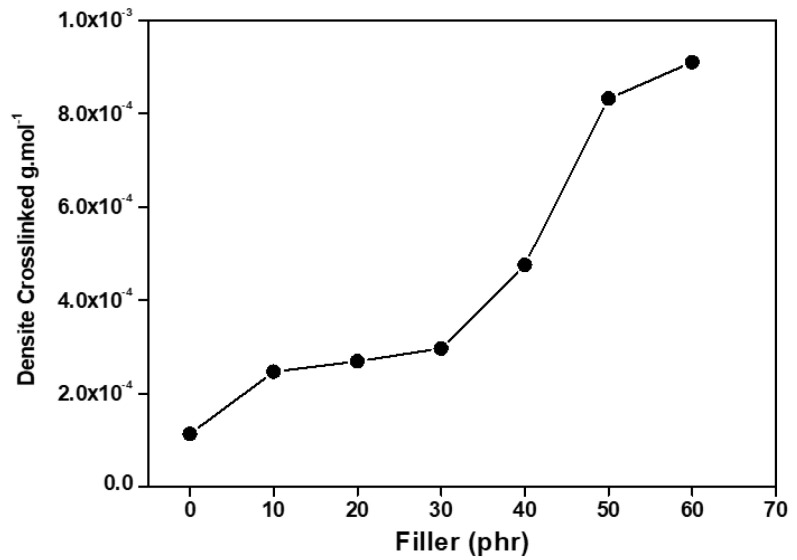
Crosslinking density of NR/LR composites.

Figure 5 illustrates the crosslinking density of the vulcanized composites containing post-industrial leather residues. A linear increase in crosslinking density is observed as the leather residue content in the samples rises, compared to vulcanized rubber without filler. This increase may be due to the formation of interfacial networks and enhanced bonding between the rubber matrix and the leather fibers.

The correlation of the number of crosslinks formed is evident in the results of the tensile strength and abrasion loss tests. Up to 20 phr of leather residue, the composites exhibited high resistance, but as the leather content increased beyond 30 phr, the stiffness compromised the flexibility of the samples, resulting in a fragile behavior. Nevertheless, this does not diminish the system’s potential for industrial applications.

### 3.3. Analysis of Rubber/Fiber Interactions Using the Lorenz–Park Equation

The interaction between the leather residue fibers and the rubber matrix was determined employing the method developed by Lorenz and Park [62] and the parameters obtained from the solvent swelling tests used in Equation (2) [21]:

(2) $$\frac{Q_{l}}{Q_{r}}={ae}^{-z}+b$$

where

- Q = the weight of toluene absorbed per gram of rubber, where subscripts l and r denote the composites vulcanized with a loading and pure rubber, respectively;
- z = the ratio of filling mass per unit mass of rubber;
- a and b = constants.

The value of Q was calculated using Equation (3):

(3) $$Q=\frac{w_{s}-w_{d}}{w_{r}\times 100/w_{F}}$$

where

- Ws = the weight of the swollen composite when the balance between the organic solvent and the polymer is achieved;
- wd = the weight of the dry composite;
- wr = the weight of the rubber in the dry composite;
- wF = the total weight of the formulation.

### 3.4. Analysis of Rubber/Loading Interactions Using the Lorentz–Park Equation

The swelling data of the composites in an organic solvent (Flory–Rehner) were used in the calculation of the Qf/Qg ratio applying Equation (2). The Qf/Qg ratio reflects the restriction to swelling by the rubber matrix in the vicinity of the loaded fibers. When rubber is immersed in the organic solvent, the latter tends to penetrate and degrade the regions that are not firmly bound to the loadings or crosslinked. Although rubber expands in response to the solvent, firmly adhered loaded particles can limit this expansion. The higher the Qf/Qg value, the weaker the load/matrix interaction because the latter tends to be load/load. And z is the ratio between the amount of loading and the amount of rubber (phr). Hence, an increase in the amount of loading in the composite generates an exponential reduction in z [63].

**Figure 6:**
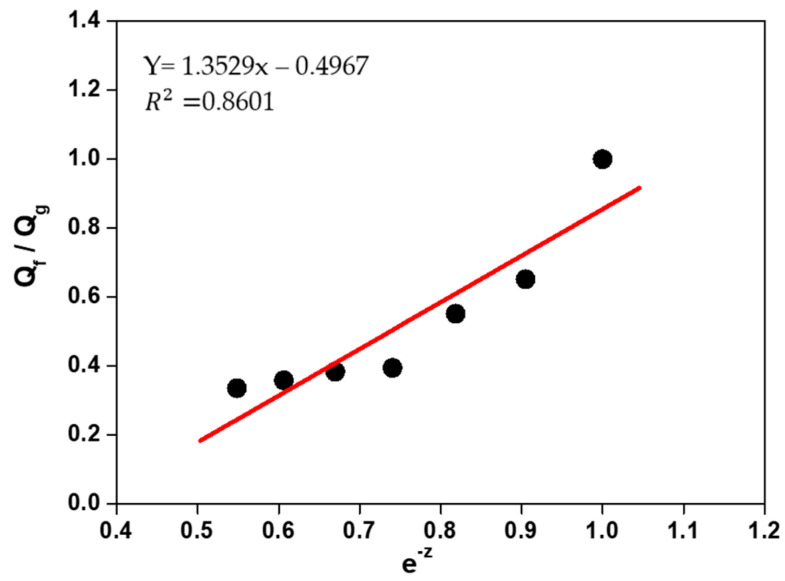
Variation in Qf/Qg versus e^−z^ in composites vulcanized with leather fiber residues.

**Figure 7:**
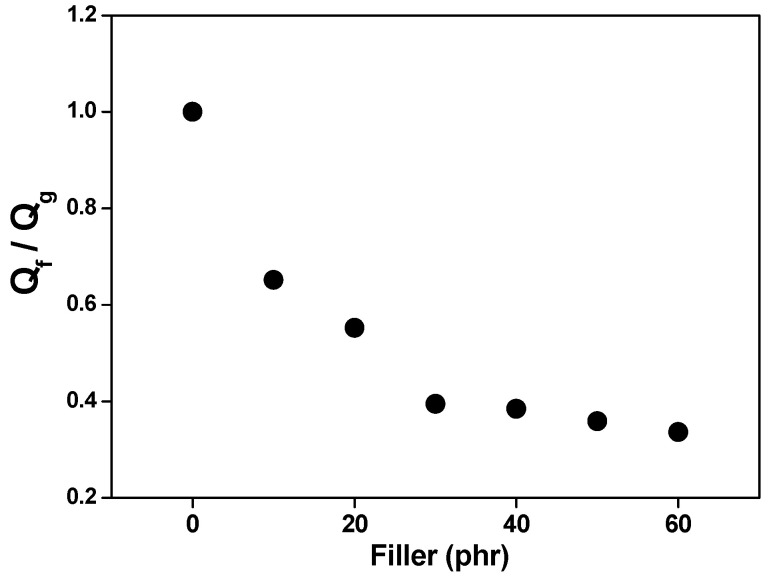
Effect of filler loading on the Qf/Qg of composites vulcanized with leather fiber residues.

Figure 5 and Figure 6 show a linear relationship between the curve of Qf/Qg and that of e^−z^. As more loading is added, the Qf/Qg ratio increases as a function of e^−z^. This behavior is due to the fact that the values are related to the swelling of the loaded rubber. Therefore, loading works as an obstruction to solvent penetration due to a good matrix/loading interaction. Parameters a and b are 1.3529 and 0.4967, respectively, with a correlation coefficient (R) of 0.8601 for leather residue fibers. According to Lorenz and Park, since the value of parameter a is greater than 0.7 and the slope of a is higher, the rubber/loading interaction is strong. The value of said parameter (much higher than 0.7) denotes a firm rubber/fiber interaction, related to the stress and strain response, respectively. Although all composites have the same formulation and crosslinking system, Figure 6 and Figure 7 show a decrease in the Qf/Qg ratio with increasing load; in other words, less solvent penetrated the more loaded composites. This is associated with improved mechanical properties and is attributed to a strong interfacial interaction between the elastomer chains and the fibers, generated by a good dispersion of said fibers in the matrix.

Figure 7 shows the Qf/Qg ratio values as a function of filler content. It can be said that the ratio values decrease as a function of the increase in the filler in the matrix, and this behavior corroborates the strong interfacial interaction between filler and rubber.

### 3.5. Relative Density

Leather’s density varies according to the stage of the production process [64]. As leather undergoes mechanical and chemical treatments, its density increases due to material compaction, which reduces, among other things, the fat content. Table 3 presents the density values of the composites as a function of the leather residue content. The addition of finished leather waste particles has been observed to proportionally increase the density of the composites. This change in density modifies physical properties such as elasticity and hardness [65], which aligns with the results of this study.

This better performance points to a competitive relationship between rubber and leather waste. The samples’ density was 10% greater than that of polybutadiene elastomers and 10% greater than that of natural leather [66], low-density EVA, medium-density soft PVC, and flexible PU, while the hardness was comparable to that of PVC, which is frequently utilized in the design of general-purpose soles [67].

### 3.6. Hardness (Shore A)

Figure 5 illustrates the crosslinking density values for the vulcanized composites. All composites display an increase in crosslinking density compared to natural rubber without filler. Shore A hardness values rise proportionally with the leather residue content in the samples. This increase can be attributed to the greater viscosity provided by the fibrous structure of leather, which is naturally stiffer than elastomeric materials [68,69].

The composite containing 60 phr of leather residue exhibited the highest crosslinking density among all samples, which coincides with the lowest swelling ratio observed. An increase in crosslinking density results in a decrease in the composite’s molecular weight, which leads to the formation of additional bridges between the collagen fibers of leather and the natural rubber matrix. Additionally, it enhances the composite’s resistance to solvents and increases the number of bonds during vulcanization. These results are consistent with those of previous studies [55,56].

**Figure 8:**
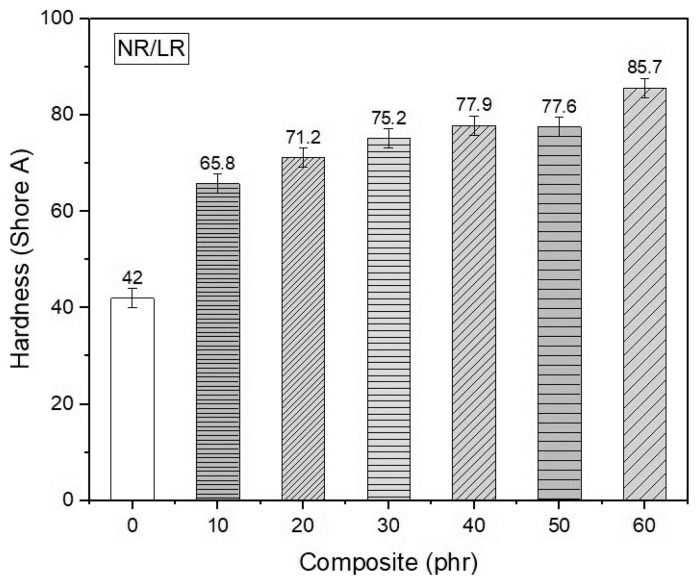
Hardness values of NR/LR composites.

The composites containing 40 and 50 phr of leather residue exhibited similar responses, shown in Figure 8. This similarity can likely be attributed to the heterogeneous dispersion of the fibers during the manual fabrication process of the samples. This suggests that a strong interphase relationship was achieved, promoting effective interaction and adhesion between the leather particles and the natural rubber matrix. When comparing the hardness parameters with the maximum torque values shown in Table 2, it is evident that both results support that the addition of leather residues increases the viscosity and hardness of the material. These findings are consistent with those reported in similar studies found in the specialized literature [55,70].

### 3.7. Abrasion Resistance

The results of the abrasion tests performed on the samples are presented in Table 4. Abrasion resistance increased for all composites, except for the one with the highest percentage of filler (60 phr), which showed a 36.8% decrease. This reduction can be attributed to structural and physical variations, such as filler–filler interactions, which may create voids in the matrix due to the high leather residue content.

The composites containing 10 and 20 phr of leather residue exhibited the highest abrasion resistance, with minimal loss volume values of 213 mm^3^ and 214 mm^3^, respectively. These results suggest the effective dispersion of the leather residues within the rubber matrix, possibly due to the method of adding fibers through sieving during the lamination process. Moreover, the incorporation of oleic acid may have improved the distribution of the leather particles and contributed to composite stability [68]. Considering that the abrasion resistance values for materials used in daily wear soles are typically < 300 mm^3^ [71], the composites containing up to 50 phr of leather residues are deemed suitable for such applications.

Other studies have demonstrated the potential to further improve abrasion resistance by incorporating higher amounts of leather residues into the rubber matrix, which may be achieved through the chemical treatment of the leather. Nevertheless, this process increases the complexity, cost, and energy consumption of the production process [71].

### 3.8. Tensile Strength/Deformation Test

**Figure 9:**
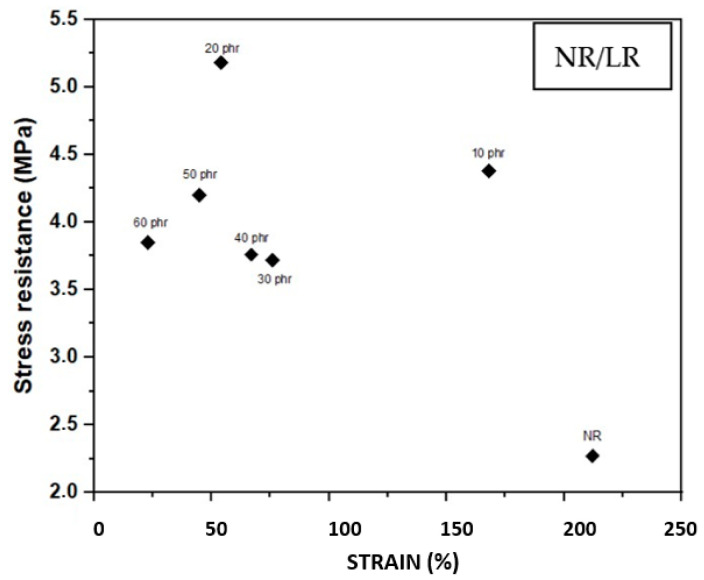
Tensile strength (stress resistance)/deformation (strain) of the NR/LR composites.

The parameters obtained from the tensile and deformation tests of the composites containing finished leather residues are presented in Table 5 and Figure 9. The results show an increase in stress values for the composites with fillers, which indicates a high level of interaction between the rubber macromolecules and the leather fibers, which are more rigid. This interaction enables the composite to withstand greater tensile stress, effectively reinforcing the material [57,72]. The small size of the leather fibers (≤600 μm) also contributes to the improved tensile strength, as smaller fibers are better dispersed within the rubber matrix, enhancing the reinforcing effect [60,65]. Notably, the composite containing 20 phr of reinforcement exhibited the highest tensile strength values (5.18 MPa), representing a 128.19% increase compared to the natural rubber without reinforcement. However, composites with more than 10 phr of leather residue demonstrated a reduction in tensile strength values compared to the NR/LR 10 composite. This decline may be attributed to the physical interference between the rubber matrix and the leather fiber chains.

This reduction in flexibility is attributed to the increased crosslinking density and stiffness of the composite. When correlating the tensile test results with the morphological analysis from the SEM images in Figure 11, particularly for samples containing 10 phr and 20 phr of leather residues, a heterogeneous and partially amorphous morphology is observed in the NR/LR 30 phr sample. These observations confirm that the reduction in ductility is consistent with both the surface morphology and the mechanical properties of the composite.

The NR/LR 10 phr composite shows a notable increase in tensile strength, with a 92.9% improvement compared to natural rubber without fillers. However, this is accompanied by a 44% decrease in deformation. The addition of leather residues increases the stiffness of the material and influences the sulfur vulcanization system, promoting the formation of polysulfide bonds that enhance the elasticity of the rubber matrix. This means that the addition of leather residues in this proportion increases the mechanical performance of the composite, making it more resistant to tensile strength (4.38 MPa) than pure natural rubber. Additionally, the composites with less than 30 phr of fibers show higher ductility than those with greater fiber content; a similar effect was found in mixtures of natural rubber with leather, but previously neutralized with chemical agents; mixtures with urea 10% had lower tensile strength, but with a higher effect in hardness, orienting this more natural compound towards products with industrial applications, including the footwear sector [71].

### 3.9. Thermogravimetric Analysis

**Figure 10:**
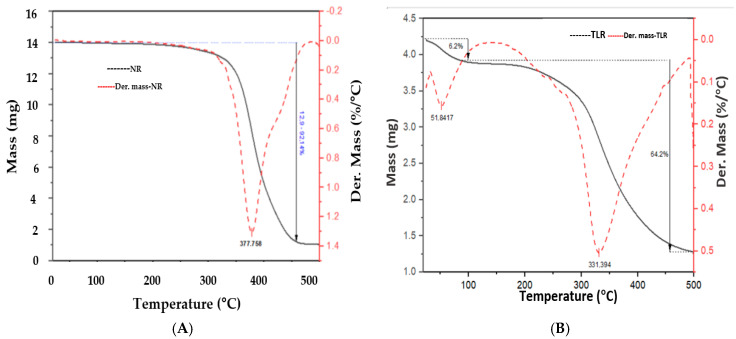
TG/DTG curves of (**A**) vulcanized natural rubber and (**B**) leather residues.

Thermogravimetric analyses were performed to assess the thermal stability of leather residues, natural rubber, and NR/LR composites through the decomposition of the material. The sample mass for this analysis ranged from approximately 14 to 23 mg. Figure 10 shows the thermogravimetry (TG) and DTG curves for (A) vulcanized natural rubber and (B) leather residue samples.

For natural rubber, a mass loss of 92.61% is observed between 280 °C and 480 °C, with an abrupt mass loss peaking at 377 °C, attributed to the degradation of the polymer chains in the rubber [73]. A lower shoulder is observed in the DTG curve (red) between 430 °C and 500 °C, characteristic of the degradation of higher-molecular-weight polymer chains, which degrade more slowly. This can also be linked to vulcanizing agents such as sulfur, which have low thermal stability and a slower degradation process [74], as observed in SEM images.

The pure natural rubber samples and the composites containing leather residues in proportions ranging from 10 to 60 phr display similar mass loss patterns, irrespective of the addition of leather residues (Figure 10A). The sample containing 60 phr of leather residues shows a mass loss of 21.6% at a maximum temperature of 300 °C (Figure 10B). This initial mass loss decreases as the leather residue content in the composite is reduced. It is noteworthy that the leather samples contained approximately 18% to 25% water, which correlates with the observed percentage of initial mass loss.

The residual mass percentage increases proportionally to the amount of leather residue in the mixture, which can be attributed to elements such as zinc oxide and sulfur from upwelling processes. However, unlike sulfur, zinc oxide requires temperatures exceeding 900 °C for degradation. Notably, the composite containing 60 phr of leather residue exhibits the lowest mass loss, with a maximum degradation temperature like that of pure natural rubber. The residual mass of this sample is higher due to the amount of leather residue, in which nitrogen is abundant, forming char and improving thermal stability.

**Figure 11:**
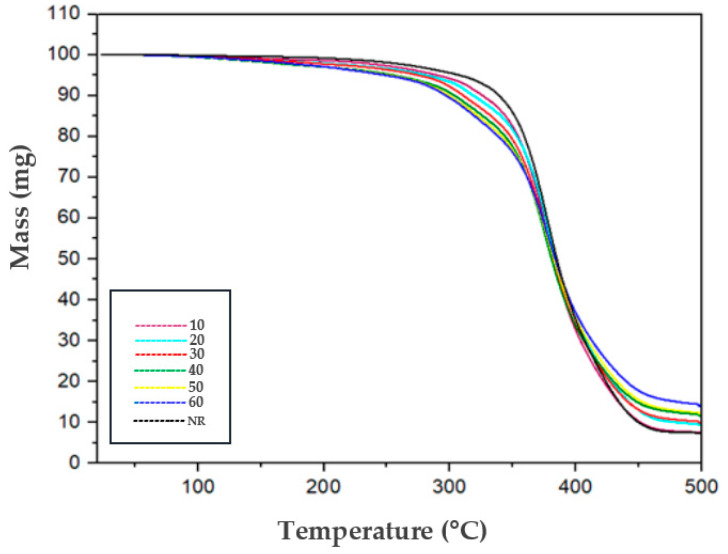
Thermogravimetric curves of the NR/LR composites.

Figure 11 demonstrates that the maximum degradation temperature for the NR/LR composites is comparable to that of the natural rubber sample without leather, suggesting a possible rubber coating that degrades at a higher temperature (378.8 °C) than leather (331.4 °C), thereby reducing mass loss and causing single-stage decomposition similar to that of natural rubber.

The degradation process of composites with higher leather residue content occurs over a wider temperature range, indicating a slower degradation rate [75]. The reduction in the intensity of the maximum degradation temperature peak and the increase in residual mass for composites containing 20 to 60 phr of leather residues demonstrate improved thermal stability. This contrasts with the composite with 10 phr and the pure natural rubber sample, which show residual masses of 7.48% and 7.39%, respectively.

In conclusion, the incorporation of leather residues through the developed methods enables the fabrication of composites with mechanical properties suited for industrial applications such as footwear and thermal insulation. Moreover, leather residues can serve not only as filler material, but also as reinforcing material, offering versatile industrial applications.

#### Morphological Evaluation: Fiber–Rubber Interaction

**Figure 12:**
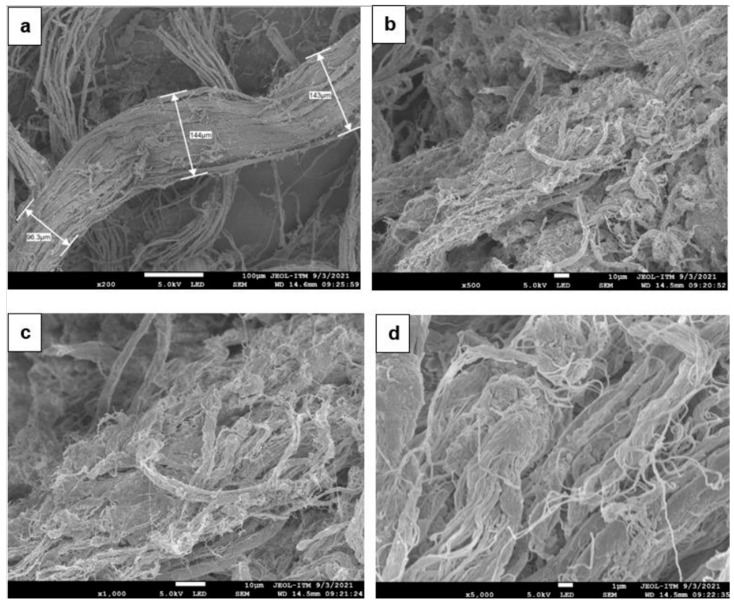
Micrographs of leather fibers at magnifications of (**a**) ×200, (**b**) ×500, (**c**) ×1000, and (**d**) ×5000.

Micrographs of the finished leather residues were randomly captured to analyze their structure and identify the relationship and distribution of the fibers prior to their incorporation into the natural rubber matrix. Figure 12 shows micrographs of leather fibers at various magnifications: (a) ×200, (b) ×500, (c) ×1000, and (d) ×5000. The interlacing of fibrils (fibrous structure) provides the material with flexibility and strength, confirming its composite nature [70,76,77].

The observed fibers have diameters ranging from 96.3 to 143 μm, as shown in Figure 12a, where individual fibrils are also visible. A 0.2 mm cross-section can contain up to 300 fibrils, each with a diameter of approximately 5 μm, and can aggregate up to 1000 filaments [78]. Figure 12b–d provide further detail of the fibrous network, revealing a matrix of intertwined fibers that form larger fibers, resulting in a high-strength material. This strength is attributed to the tanning process with chromium (III), which reacts with the amino acid chains to stabilize collagen, contributing good physical properties to the material [79].

The fibers are separated and well defined as a result of the tanning process applied to the material, which enhances its mechanical properties and makes it suitable for use in industry. This process, which improves the material softness, flexibility, and permeability, also results in the isolation of fibrils through the creation of voids that render the structure more porous and striated [80]. These properties improve the interaction of the rubber matrix with the leather fibers and are characteristic of low-density materials.

**Figure 13:**
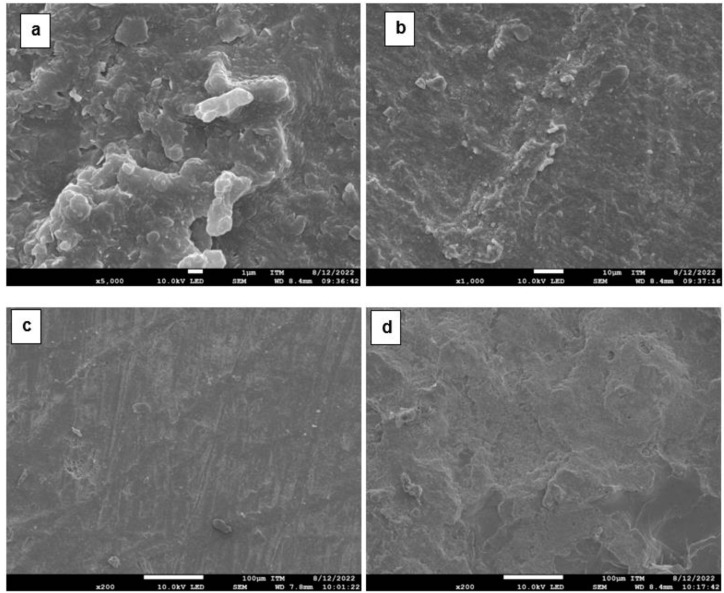
Composite micrographs: (**a**) NR/LR 10 at ×5000, (**b**) NR/LR 10 at ×1000, (**c**) NR/LR 20 at ×200, (**d**) NR/LR 30 at ×200.

Figure 13 presents micrographs of the composites with different filler proportions: (a and b) 10 phr, (c) 20 phr, and (d) 30 phr. The composites exhibit a predominant dark coloration, which is consistent across all samples regardless of the amount of leather added. This darkening can be explained by the natural color of the filler as well as the heating process, which causes the fibers to become more opaque due to dehydration. There is also evidence of increased surface roughness; however, no exposed leather fibers are observed, indicating the good coverage of the leather fibers by the rubber matrix.

**Figure 14:**
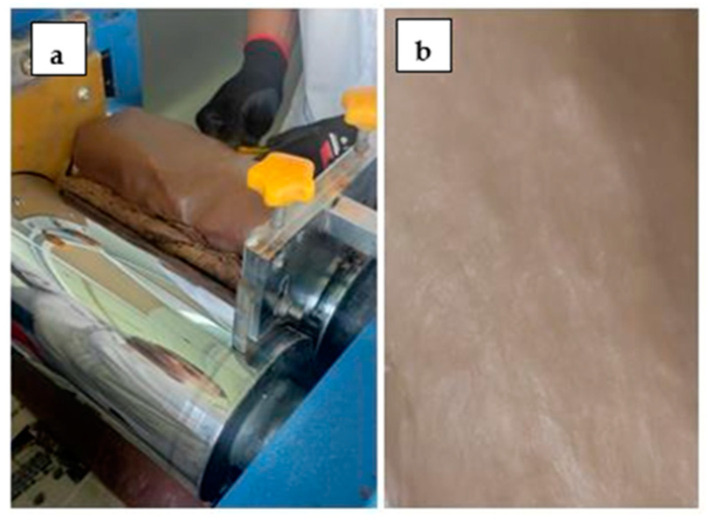
Composite obtained.

The composite containing 20 phr fibers, shown in Figure 13c, displays a smoother surface compared to that in images a and b. This difference is due to the texture of the aluminum plate used during thermoforming, whereas the two first images represent cross-sectional views. In Figure 14, image (a) shows the process of mixing rubber with recycled leather and image (b) shows the compound obtained with 10 phr of material; as can be seen, it is a homogeneous product, which will have many applications in the footwear manufacturing sector, for example.

## 4. Final Observations

The reuse of leather waste for the development of new products through the traditional process of mixing rubber in roller cylinders, as outlined and proposed at the beginning of this article, has the potential to contribute significantly to the adoption of sustainable technologies. By collaborating with companies from other sectors and replicating programs from developed countries, this approach can become a driving force in transforming a highly productive sector, aligning it with sustainable practices and clean production methods.

By-products such as trimmings, sawdust, and shavings, which originate from the tanning and manufacturing processes, are currently considered non-usable waste by the company and are consequently disposed of with solid waste in a landfill. In contrast, wet blue leather, due to its chemical content, is separately disposed of by an external company. However, creating partnerships between the tannery and regional craft sector associations would offer the possibility for mutual benefits: the tannery could dispose of its leather waste and the craft sector would gain access to valuable raw materials.

## 5. Conclusions

A sustainability program must be a continuous effort rather than a one-time initiative. Once a plan is put in place, it is imperative to identify improvements that prioritize both environmental and financial sustainability. This is particularly important for Colombia’s leather tanning companies, which are predominantly family-owned businesses with limited or inexistent environmental awareness in their practices.

It is crucial to inform small and medium-sized producers about available alternatives for utilizing waste through tools like clean production and design engineering. This study demonstrates that it is possible to carry out collaborative processes across different production areas, contributing to the achievement of Sustainable Development Goals 12 (Responsible Consumption and Production), 9 (Industry, Innovation, and Infrastructure), and 17 (Partnership for the Goals). Moreover, these efforts strengthen the communities where they take place.

To boost sustainability in Colombia’s leather tanning industry, ongoing education for small producers is vital. Promoting waste utilization and exploring innovative materials with leather waste and natural rubber will enhance clean practices and community resilience.

Utilizing leather waste with alternative materials opens up opportunities to create new and innovative materials. Mixing leather waste with natural rubber provides a sustainable option for creating value-added products with positive environmental impact.

## Figures and Tables

**Table 1:** Vulcanization system formulation (phr).

Function	Materials	phr
Matrix	Natural rubber (NR)	100
Filler	Leather residue (LR)	0, 10, 20, 30, 40, 50, 60
Activator	Zinc oxide (ZnO)	5
Activator	Stearic acid	2
Curing agent	Sulfur (S)	2.5
Lubricant	* Oleic acid	6
Secondary accelerator	** TMTM	0.5
Primary accelerator	*** MBTS	1.25

* (9Z)-9-octadecenoic acid; ** Tetramethylthiuram monosulfide; *** Dibenzothiazole disulfide.

**Table 2:** Rheometric parameters of NR/LR composites.

Composite (100 phr)	MH (dNm)	ML (dNm)	ΔM (dNm)	t90 (dNm)
NR 0	0.68 ± 0.12	0.05 ± 0.12	0.63 ± 0.12	6.336 ± 0.12
NR/LR 10	0.88 ± 0.12	0.09 ± 0.14	0.79 ± 0.13	9.65 ± 0.12
NR/LR 20	1.27 ± 0.13	0.12 ± 0.15	1.15 ± 0.14	10.618 ± 0.14
NR/LR 30	1.03 ± 0.14	0.14 ± 0.13	0.89 ± 0.11	8.952 ± 0.13
NR/LR 40	1.49 ± 0.13	0.24 ± 0.11	1.25 ± 0.12	8.216 ± 0.13
NR/LR 50	1 ± 0.14	0.2 ± 0.12	0.8 ± 0.13	6.554 ± 0.12
NR/LR 60	1.77 ± 0.13	0.26 ± 0.11	1.51 ± 0.11	6.098 ± 0.14

**Table 3:** Density for NR/LR composites.

Composite (phr)	Density (g/cm^3^)
Pure gum (NR 0)	0.98 ± 2
NR/LR 10	1.0 ± 2
NR/LR 20	1.01 ± 2
NR/LR 30	1.02 ± 2
NR/LR 40	1.04 ± 3
NR/LR 50	1.06 ± 3
NR/LR 60	1.07 ± 3

**Table 4:** Abrasion resistance.

Composite (phr)	Loss Volume (mm^3^)	Abrasion Resistance (%)
NR	264.2 ± 2.4	59 ± 3
NR/LR 10	214.2 ± 1.4	72.6 ± 2
NR/LR 20	213.6 ± 2.8	72.9 ± 2
NR/LR 30	251.6 ± 9.6	61.9 ± 3.3
NR/LR 40	245.5 ± 8.4	63.5 ± 2.3
NR/LR 50	258.4 ± 8.9	60.4 ± 3.9
NR/LR 60	422.5 ± 7.6	36.8 ± 2.3

**Table 5:** Tensile strength/deformation test results.

Composite (phr)	Tensile Strength (MPa)	Deformation (%)
NR	2.27 ± 0.41	212 ± 0.39
NR/LR 10	4.38 ± 1.16	168 ± 1.12
NR/LR 20	5.18 ± 0.27	54 ± 0.08
NR/LR 30	3.72 ± 0.69	76 ± 0.46
NR/LR 40	3.76 ± 0.86	67 ± 0.15
NR/LR 50	4.2 ± 0.4	45 ± 0.08
NR/LR 60	3.85 ± 0.29	23 ± 0.05

## Data Availability

The original contributions presented in this study are included in the article. Further inquiries can be directed to the corresponding authors.
